# Momentum space separation of quantum path interferences between photons and surface plasmon polaritons in nonlinear photoemission microscopy

**DOI:** 10.1515/nanoph-2023-0776

**Published:** 2024-03-05

**Authors:** Pascal Dreher, David Janoschka, Harald Giessen, Ralf Schützhold, Timothy J. Davis, Michael Horn-von Hoegen, Frank-J. Meyer zu Heringdorf

**Affiliations:** Faculty of Physics and Center for Nanointegration, Duisburg-Essen (CENIDE), 27170 University of Duisburg-Essen, 47048 Duisburg, Germany; 9149 4th Physics Institute, Research Center SCoPE, and Integrated Quantum Science and Technology Center, University of Stuttgart, 70569 Stuttgart, Germany; School of Physics, University of Melbourne, Parkville, Victoria 3010, Australia; Helmholtz-Zentrum Dresden-Rossendorf, Bautzner Landstrasse 400, 01328 Dresden, Germany; Institut für Theoretische Physik, Technische Universität Dresden, 01062 Dresden, Germany; University of Duisburg-Essen, 47057 Duisburg, Germany

**Keywords:** PEEM, surface plasmon polaritons, photoemission

## Abstract

Quantum path interferences occur whenever multiple equivalent and coherent transitions result in a common final state. Such interferences strongly modify the probability of a particle to be found in that final state, a key concept of quantum coherent control. When multiple nonlinear and energy-degenerate transitions occur in a system, the multitude of possible quantum path interferences is hard to disentangle experimentally. Here, we analyze quantum path interferences during the nonlinear emission of electrons from hybrid plasmonic and photonic fields using time-resolved photoemission electron microscopy. We experimentally distinguish quantum path interferences by exploiting the momentum difference between photons and plasmons and through balancing the relative contributions of their respective fields. Our work provides a fundamental understanding of the nonlinear photon–plasmon–electron interaction. Distinguishing emission processes in momentum space, as introduced here, could allow nano-optical quantum-correlations to be studied without destroying the quantum path interferences.

## 1 Introduction

Quantum path interference occurs when multiple coherent pathways can take a system from an initial state to a final state, as epitomized in Feynman’s path-integral interpretation [1]. In the last century, Tannor & Rice [2] and Brumer & Shapiro [3] realized that coherent light can be used to create quantum path interferences in matter. Coherent control of such quantum path interferences has been experimentally demonstrated for numerous physical systems including photoexcitation and -ionization of atoms [4], [5], [6], [7] and molecules [8], [9], [10], [11], [12], the interaction of free electrons with light [13], [14], [15], photoinduced structural dynamics [16], [17] and nonlinear photoemission [18], [19], [20], [21] at surfaces. In all these cases, quantum path interference relies on not knowing which path a system takes. Measurements aiming at obtaining this information destroy the interference, as observed in “which-way” experiments [22].

Nonlinear electron emission at surfaces can also be triggered by the absorption of surface plasmon polaritons (SPPs) [23], [24], [25], [26], [27], [28], in similarity to the absorption of light [29]. Microscopically, SPPs are a quantized (collective) many-body excitation of the electron system. It has been demonstrated that coherent control of SPP excitation [30] and SPP-triggered electron emission [31] is possible. A particularly interesting situation arises in electron emission during the *simultaneous* presence of light and SPPs, where the absorption of energy-degenerate quanta from both fields can create multiple quantum pathways [32], [33], [34]. As these pathways result in a common final state of the liberated electron, quantum path interferences appear in the electron yield [35], [36], [37], [38].

The multitude of possible nonlinear interactions that form the different pathways during simultaneous light and SPP absorption makes it difficult to experimentally disentangle the resulting quantum path interferences. Here, in analogy to optical nonlinear spectroscopy [39], [40], we use a novel momentum space approach to separate quantum path interferences in nonlinear photoemission electron microscopy (PEEM).

## 2 Experimental details

**Figure 1:**
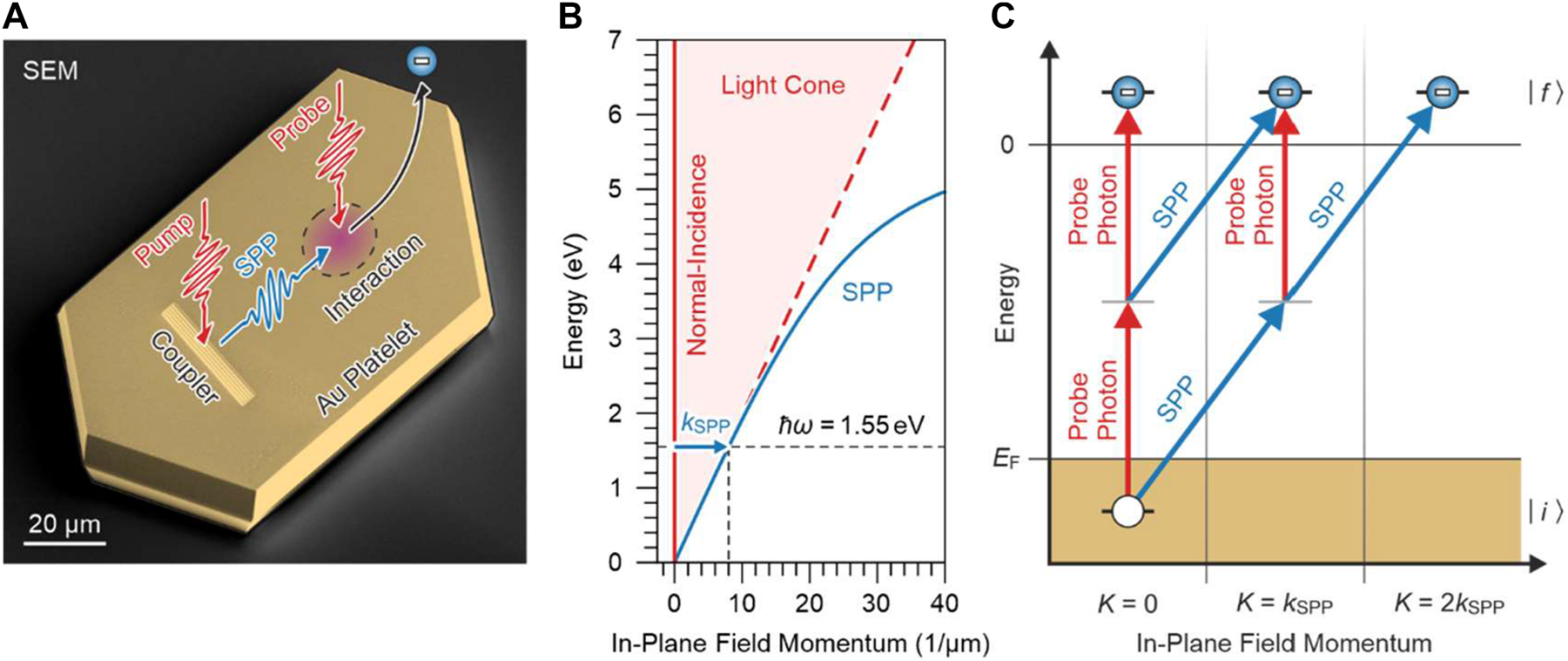
Mixing of SPPs and light in electron emission. (A) Sketch of the utilized pump–probe scheme. The scanning electron micrograph shows a platelet similar to the one used for the presented experiments. The arrows illustrate the different light and SPP pulses. (B) Dispersion relation for SPPs and light as a function of the momentum in the surface plane. It is the momentum mismatch between normally incident light and the SPPs that we exploit to distinguish individual quantum path interferences. (C) Energy-level diagram of the different couplings that can occur in the interaction region of SPPs and probe photons. The different states and couplings are sorted by the in-plane momentum transfer during the electron emission.

The experiments were performed in a spectroscopic photoemission and low-energy electron microscope (ELMITEC SPE-LEEM III) [41] equipped with a highly sensitive and linear electron detector [42]. The microscope is combined with a Ti:Sapphire oscillator (Femtolasers Femtosource Compact) that provides us with <15 fs laser pulses with a central wavelength of 800 nm $\left(\hbar \omega =1.55\;\text{eV}\right)$ at a repetition rate of 80 MHz. We worked in a normal-incidence geometry [43] and used a Pancharatnam’s phase stabilized Mach–Zehnder interferometer [44], [45] to create pairs of mutually delayed pump and probe laser pulses with sub-femtosecond accuracy. The setup is similar to the one used in Ref. [46]. Half-wave plates in each of the two arms of the interferometer in combination with a Brewster polarization plate at the output of the interferometer were used to independently tune the power of the pump and the probe laser pulses while maintaining a common linear polarization axis. Before the laser pulses entered the microscope, the final linear polarization was adjusted to be perpendicular to a grating coupler on the sample (cf. Figure 1A) with another, freely adjustable, half-wave plate.

The grating coupler was cut into a single-crystalline Au(111) platelet [47] by focused ion beam milling (FIB) using a FEI Helios NanoLab 600. The sample was transferred through air into the microscope and subsequently cleaned by *in situ* oxygen plasma etching, Argon ion sputtering, and degassing at elevated temperature in ultra-high vacuum. Prior to the photoemission experiments, we lowered the work-function of the sample by deposition of a sub-monolayer of Cs from a commercial dispenser (SAES Getters) to enable a second-order electron emission process.

## 3 Results

An overview of the experiment is shown in Figure 1A. A first femtosecond laser pulse (pump) excites an SPP pulse at the grating coupler. We do not take the conversion of the pump pulse into an SPP pulse by the grating coupler into consideration and instead treat the SPP as an independent propagating polarization field. After the SPP has freely propagated for about 80 fs, a subsequent laser pulse (probe) arrives at the surface and forms an interaction region with the SPP pulse. At this time, the pump laser pulse has already decayed. Thus, only the combined SPP and probe pulse initiate second-order absorption liberating an electron from the surface.

A simple measurement of the electron emission yield from the interaction region provides no information about from which of the two fields quanta are absorbed. However, the different propagation directions of the SPP pulse and the probe laser pulse at the metal surface result in a significant momentum difference between them (Figure 1B). As such, despite being degenerate in energy, during absorption different combinations of SPPs and probe photons are associated with different in-plane field momenta [39], [48], [49]. Figure 1C summarizes the possible couplings between electronic states during electron emission due to the interaction with the SPP and probe field sorted by the associated in-plane field momenta. Naively, from these couplings one might expect that in lowest emission order an electron could be liberated by either the consecutive absorption of two photons from the probe field, the consecutive absorption of an SPP and a probe photon, or the consecutive absorption of two SPPs. As the key concept of the current work, we will show that the involved field momenta provide an electron emission signature that enables us to experimentally identify and separate quantum path interferences that arise from the different couplings.

**Figure 2:**
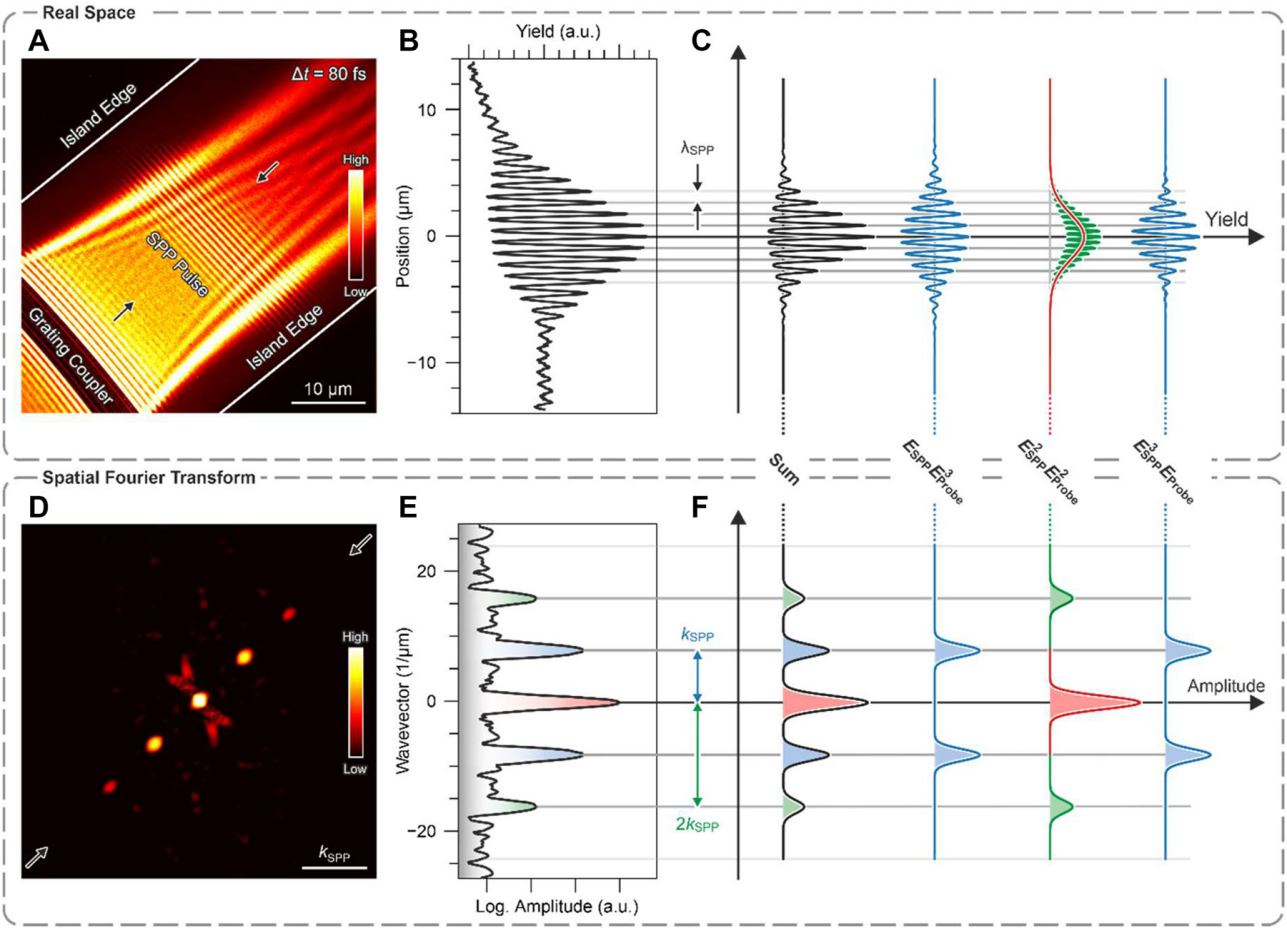
Fourier decomposition of SPP-light correlations in PEEM. (A) Time-resolved PEEM image of an SPP pulse 80 fs after excitation at the grating coupler, depicted in a linear false-color scale. The fringes in the center of the image are a direct conceptual visualization of the propagating SPP pulse. (B) Section through the fringe-pattern in the electron yield distribution in real-space as indicated by the arrows in (A). (C) Sketch of the contribution of the different terms in Eq. (4) to the spatial fringe-pattern in the electron yield (B) sorted by powers in the SPP and the probe field. (D) Wavevector spectrum computed via a windowed Fourier transform of an electron-optically magnified image of the interaction region in (A) depicted on a logarithmic false-color scale. The five visible peaks arise from the fringe modulation in real space, and their equidistant spacing is given by the SPP wavenumber. (E) Section through the SPP wavevector spectrum as indicated by the arrows in (D). (F) Sketch of the contribution of the different terms in Eq. (4) to the wavevector spectrum in (E) sorted by powers in the SPP and the probe field.

The interaction region formed by the SPP and the probe laser pulse appears in the PEEM image as a spatial fringe modulation (Figure 2A), which is a signature of the propagating SPP pulse at this particular pump–probe delay. In the classical field picture, this characteristic electron emission pattern [43], [50], [51] is due to the interference of the SPP and the probe laser field. In a quantum description, such a fringe modulation must be attributed to quantum path interferences in the electron emission process, as shown in a recent experiment [52] on spin–orbit mixing of SPPs with orbital angular momentum [53] and circularly polarized light. Since the period length of the fringe pattern is determined by the SPP wavelength *λ*_SPP_, the pattern is commonly referred to as a “direct conceptual visualization” of the SPP pulse [43], [54]. A profile taken through the fringe pattern, however, shows a distinct nonlinearity (Figure 2B), which appears as a second-order cross-correlation of the pulses. In Figure 2C, we decompose this profile into contributions arising from the featureless envelopes of the pulses, contributions with periods equal to the SPP wavelength, and one contribution with half the SPP wavelength. These contributions to the nonlinear profile are a direct manifestation of different quantum path interferences that occur in the electron emission.

To understand the origin of the quantum path interferences, we first consider the electron emission probability amplitude *S*_*fi*_ from the couplings depicted in Figure 1C. For standard photoemission, the coupling is usually described as a dipolar coupling of the involved electronic states to a classical light field. In contrast, the SPP is a quantized many-body excitation of the electron system itself, consisting of the hybridization of a surface plasmon, i.e., a coherent sum of electron–hole pair excitations, with a photon [55], [56], [57]. In the case of SPP-driven electron emission, the coupling of SPPs to the single-electron states involved in the emission appears to be similar to the case of light [29] and can also be described as a dipolar coupling [55], [58]. Accordingly, if the electronic part of the SPP is neglected, a quantization of the evanescent electromagnetic field of the SPP [58], [59] is approximately sufficient to describe the emission. To arrive at a consistent description of the electron emission, we thus quantize the electromagnetic field at the sample’s surface and treat both, the SPPs and the probe photons, equally as independent quantized modes thereof. Note that a quantization of the probe field is not strictly necessary to describe the experimental signatures here but aids in understanding these signatures in a particle picture in terms of quantal absorptions of SPPs and photons by the electron system.

As we will show, the quantal absorption processes are combined to form the discussed quantum path interferences during electron emission. Neglecting the already decayed pump laser pulse, the electron emission requires the absorption of at least two quanta from a joint initial state $\left|\psi \right\rangle$ of the probe field and the SPP, arriving in a joint final state $\left|\phi \right\rangle$ of these fields. Similar to the theory of two-photon absorption by Mollow [60], assuming weak dipole couplings, we expand the electron emission probability amplitude *S*_*fi*_ in the dominant emission order using second-order perturbation theory in the quantized fields and arrive at

(1) $$\begin{array}{ll} S_{fi}\left(R,\Delta t\right) & =\iint \text{d}t_{1}\text{d}t_{2}L_{fi}\left(t_{1},t_{2}\right)\\ & \;\times \left\langle \phi \right|\left({\hat{E}}_{\text{Probe}}^{\left(+\right)}\left(t_{2}-\Delta t\right)+{\hat{E}}_{\text{SPP}}^{\left(+\right)}\left(R,t_{2}\right)\right)\\ & \;\times \left({\hat{E}}_{\text{Probe}}^{\left(+\right)}\left(t_{1}-\Delta t\right)\right)+{\hat{E}}_{\text{SPP}}^{\left(+\right)}\left(R,t_{1}\right)\left|\psi \right\rangle \end{array}$$

where **R** is the position on the surface and Δ*t* is the pump–probe delay. The details of the second-order response of the electron system and in particular the associated dipole moments are contained in the two-time dipole correlation function $L_{fi}\left(t_{1},t_{2}\right)$ [60]. We focus on the quantum transition of the fields, which is due to the annihilation of photons and SPPs by the positive-frequency field operators ${\hat{E}}_{\text{Probe}}^{\left(\;+\;\right)}\left(t\right)$ and ${\hat{E}}_{\text{SPP}}^{\left(\;+\;\right)}\left(R,t\right)$ . Only the SPP operator depends on the spatial coordinate **R** due to the normal incidence of the laser pulses [43]. On expanding the terms in Eq. (1), we can find all time-ordered combinations of consecutive interactions with both fields, such as ${\hat{E}}_{\text{Probe}}^{\left(+\right)}{\hat{E}}_{\text{Probe}}^{\left(+\right)}$ , ${\hat{E}}_{\text{SPP}}^{\left(+\right)}{\hat{E}}_{\text{Probe}}^{\left(+\right)},{\hat{E}}_{\text{Probe}}^{\left(+\right)}{\hat{E}}_{\text{SPP}}^{\left(+\right)}$ , and ${\hat{E}}_{\text{SPP}}^{\left(+\right)}{\hat{E}}_{\text{SPP}}^{\left(+\right)}$ , which have been indicated in Figure 1C.

The overall probability *P* for a second-order electron emission process is given by the magnitude square of the probability amplitude *S*_*fi*_ summed over all final states of the fields and over all possible initial and final electronic states. We arrive at

(2) $$\begin{array}{ll} P\left(R\right) & =∭\;\int \text{d}t_{1}^{\prime }\text{d}t_{2}^{\prime }\text{d}t_{1}\text{d}t_{2}M\left(t_{1}^{\prime },t_{2}^{\prime };t_{1},t_{2}\right)\\ & \;\times E\left(R;t_{1}^{\prime },t_{2}^{\prime };t_{1},t_{2}\right) \end{array}$$

where the dipole correlation functions are absorbed into the response function M as discussed in the Supplementary Note 1. While the response function M can in principle capture excited electrons and thus long-lived correlations, we assume it to be instantaneous and only consider equal-time interactions of the electron system with the excited SPPs and the probe photons. This is motivated by the absence of real intermediate states in the sp-band gap of the Au(111) surface along the Γ − *L* direction [61] such that only virtual intermediate states can facilitate a second-order electron emission process. Furthermore, at the moment of emission, the electron system is considered to be in a thermalized ground state because of the short relaxation times of electrons excited by one pump photon above the Fermi edge in Au(111) [62], [63]. Considering the 80 fs large pump–probe delay in our experiment and the substantially smaller relaxation time, we can safely assume that effects from a transient population induced by the pump can be neglected.

The electron emission probability *P* in Eq. (2) essentially depends on the second-order coherence function E of the field operators (see Supplementary Note 1) with

(3) $$\begin{array}{ll} E\left(R;t_{1}^{\prime },t_{2}^{\prime };t_{1},t_{2}\right) & =\left\langle \left({\hat{E}}_{\text{Probe}}^{\left(-\right)}\left(t_{1}^{\prime }-\Delta t\right)+{\hat{E}}_{\text{SPP}}^{\left(-\right)}\left(R,t_{1}^{\prime }\right)\right)\right.\\ & \;\times \left({\hat{E}}_{\text{Probe}}^{\left(-\right)}\left(t_{2}^{\prime }-\Delta t\right)+{\hat{E}}_{\text{SPP}}^{\left(-\right)}\left(R,t_{2}^{\prime }\right)\right)\\ & \;\times \left({\hat{E}}_{\text{Probe}}^{\left(+\right)}\left(t_{2}-\Delta t\right)+{\hat{E}}_{\text{SPP}}^{\left(+\right)}\left(R,t_{2}\right)\right)\\ & \;\times {\left.\left({\hat{E}}_{\text{Probe}}^{\left(+\right)}\left(t_{1}-\Delta t\right)+{\hat{E}}_{\text{SPP}}^{\left(+\right)}\left(R,t_{1}\right)\right)\right\rangle }_{\hat{\sigma }}. \end{array}$$

Here, ${\left\langle \ldots \right\rangle }_{\hat{\sigma }}=\mathrm{Tr}\left(\hat{\sigma }\ldots \right)$ denotes an expectation value with respect to the joint initial state density matrix $\hat{\sigma }$ of the probe field and the SPP field, and ${\hat{E}}_{i}^{\left(-\right)}={\left({\hat{E}}_{i}^{\left(+\right)}\right)}^{†}$ are the negative-frequency field (i.e., creation) operators. The second-order electron emission probability depends on fourth-order interactions with the probe and the SPP field, i.e., on fourth-order products of annihilation and creation operators, as in cross-terms like ${\hat{E}}_{\text{Probe}}^{\left(-\right)}{\hat{E}}_{\text{Probe}}^{\left(-\right)}{\hat{E}}_{\text{Probe}}^{\left(+\right)}{\hat{E}}_{\text{SPP}}^{\left(+\right)}$ . These cross-terms represent the quantum path interferences that are formed by the different couplings sketched in Figure 1C.

The spatial fringe modulation with the SPP wavevector **k**_SPP_ observed in the electron yield is obtained by considering the (approximate) plane-wave nature of the SPP in the surface plane ${\hat{E}}_{\text{SPP}}^{\left(\pm \right)}\propto \text{exp}(\pm ik_{\text{SPP}}\cdot R)$ . The terms involving products of dissimilar operators, ${\hat{E}}_{\text{SPP}}^{\left(\pm \right)}{\hat{E}}_{\text{SPP}}^{\left(\mp \right)}$ , are independent of the SPP wavevector, those involving just one SPP field operator ${\hat{E}}_{\text{SPP}}^{\left(\pm \right)}$ depend on $\text{exp}(\pm ik_{\text{SPP}}\cdot R)$ , and the terms involving only products of similar operators, ${\hat{E}}_{\text{SPP}}^{\left(\pm \right)}{\hat{E}}_{\text{SPP}}^{\left(\pm \right)}$ , depend on $\text{exp}(\pm i2k_{\text{SPP}}\cdot R)$ . On this basis, the measured electron emission yield profile of Figure 2B can be decomposed into the different contributions of Figure 2C. They arise from different mixings of the fields during electron emission, which in turn depend on integer multiples of the SPP wavevector.

To clarify which of the contributions to the electron yield must be interpreted as quantum path interferences, we obtain the quantum-mechanical electron emission rate by expanding Eq. (2) in a momentum space that is spanned by the real-space periodic modulations of the electron yield. This momentum space must not be confused with the momentum space spanned by the emission angles of the liberated electrons. As derived in Supplementary Note 1 for the aforementioned approximations, the resulting electron emission rate in momentum space is

(4) $$\begin{array}{ll} \Gamma_{2\text{PPE}}^{\text{quantum}}\left(K,\Delta t\right) & \propto \delta \left(K\right)\left(\frac{{\langle \hat{a}}^{†}{\hat{a}}^{†}\hat{a}{\hat{a}\rangle }_{\hat{\sigma }}}{N_{\text{Probe}}^{2}}+\varepsilon_{\text{SPP}}^{4}\frac{{\langle \hat{b}}^{†}{\hat{b}}^{†}\hat{b}{\hat{b}\rangle }_{\hat{\sigma }}}{N_{\text{SPP}}^{2}}\right)\\ & \;+\delta \left(K\right)\frac{1}{N_{\text{Probe}}N_{\text{SPP}}}\left({\langle \hat{a}}^{†}{\hat{b}}^{†}\hat{a}{\hat{b}\rangle }_{\hat{\sigma }}+{\langle \hat{b}}^{†}{\hat{a}}^{†}\hat{b}{\hat{a}\rangle }_{\hat{\sigma }}+\varepsilon_{\text{SPP}}^{2}\left({\langle \hat{b}}^{†}{\hat{a}}^{†}\hat{a}{\hat{b}\rangle }_{\hat{\sigma }}+{\langle \hat{a}}^{†}{\hat{b}}^{†}\hat{b}{\hat{a}\rangle }_{\hat{\sigma }}\right)\right)\\ & \;+\delta \left(K-k_{\text{SPP}}\right)\frac{e^{-i\omega \Delta t}}{\sqrt{N_{\text{Probe}}N_{\text{SPP}}}}\left(\frac{{\langle \hat{a}}^{†}{\hat{a}}^{†}\hat{a}{\hat{b}\rangle }_{\hat{\sigma }}+{\langle \hat{a}}^{†}{\hat{a}}^{†}\hat{b}{\hat{a}\rangle }_{\hat{\sigma }}}{N_{\text{Probe}}}+\varepsilon_{\text{SPP}}^{2}\frac{{\langle \hat{a}}^{†}{\hat{b}}^{†}\hat{b}{\hat{b}\rangle }_{\hat{\sigma }}+{\langle \hat{b}}^{†}{\hat{a}}^{†}\hat{b}{\hat{b}\rangle }_{\hat{\sigma }}}{N_{\text{SPP}}}\right)\\ & \;+\delta \left(K+k_{\text{SPP}}\right)\frac{{\text{e}}^{\text{i}\omega \Delta t}}{\sqrt{N_{\text{Probe}}N_{\text{SPP}}}}\left(\frac{{\langle \hat{b}}^{†}{\hat{a}}^{†}\hat{a}{\hat{a}\rangle }_{\hat{\sigma }}+{\langle \hat{a}}^{†}{\hat{b}}^{†}\hat{a}{\hat{a}\rangle }_{\hat{\sigma }}}{N_{\text{Probe}}}\right.\left.+\varepsilon_{\text{SPP}}^{2}\frac{{\langle \hat{b}}^{†}{\hat{b}}^{†}\hat{b}{\hat{a}\rangle }_{\hat{\sigma }}+{\langle \hat{b}}^{†}{\hat{b}}^{†}\hat{a}{\hat{b}\rangle }_{\hat{\sigma }}}{N_{\text{SPP}}}\right)\\ & \;+\delta \left(K-2k_{\text{SPP}}\right)e^{-2i\omega \Delta t}\frac{{\langle \hat{a}}^{†}{\hat{a}}^{†}\hat{b}\hat{b}\rangle {}_{\hat{\sigma }}}{N_{\text{Probe}}N_{\text{SPP}}}+\delta \left(K+2k_{\text{SPP}}\right)e^{2i\omega \Delta t}\frac{{\langle \hat{b}}^{†}{\hat{b}}^{†}\hat{a}{\hat{a}\rangle }_{\hat{\sigma }}}{N_{\text{Probe}}N_{\text{SPP}}}. \end{array}$$

Here, **K** is the wavevector in the surface plane and $\varepsilon_{\text{SPP}}^{2}$ is the squared magnitude of the SPP polarization vector. The field operators have been decomposed into annihilation and creation operators for the probe photons, $\hat{a}$ and ${\hat{a}}^{†}$ , and likewise for the SPPs, $\hat{b}$ and ${\hat{b}}^{†}$ , where $N_{\text{Probe}}$ and $N_{\text{SPP}}$ are the associated normalization constants.

While Eq. (4) consists of 16 different fourth-order equal-time initial state correlation functions of the SPP and the probe field, only 6 of these correlation functions are independent. The correlation functions for positive and negative wavevectors, i.e., the correlation functions in the third and fourth line of Eq. (4) as well as the two correlation functions in the last line of Eq. (4), are identical up to complex conjugation. Moreover, some of the remaining correlation functions are identical up to equal-time commutations of the involved operators. Each of these correlation functions is interpreted as an individual electron emission pathway in Liouville space [64], and we will identify which of these pathways correspond to quantum path interferences in the context of Figure 4.

First, however, we compare our experiment to Eq. (4) and demonstrate the existence of all of the 6 independent Liouville pathways in the experimental data. For this purpose, we calculate the wavevector spectrum of the data (Figure 2D and E) via a spatial Fourier transformation of the PEEM image of Figure 2A. The wavevector spectrum consists of 5 distinct peaks at multiples of the SPP wavenumber $\left|k_{\text{SPP}}\right|=2\pi /\lambda_{\text{SPP}}$ , located on a line perpendicular to the SPP phase fronts. The first-order peaks at **K** = ±**k**_SPP_ correspond to the fringe modulation of the electron yield in real space with periodicity *λ*_SPP_, justifying the usual interpretation of the fringe pattern as a “direct conceptual visualization” of the SPP pulse. The second-order peaks at **K** = ±2**k**_SPP_, however, correspond to a periodic modulation of the electron yield at half the SPP wavelength and are a direct consequence of the nonlinear emission process. We do not observe third-order peaks at **K** = ±3**k**_SPP_, which corroborates that electron emission orders higher than second-order are negligible and the perturbative derivation of the theory outlined above is justified.

Each of the different Liouville pathways in Eq. (4) is characterized by a distinct wavevector **K**, a distinct harmonic delay-dependence, and a distinct dependence on the product of annihilation and creation operators. The quantum-mechanical transition rate closely resembles the structure of the common phenomenological model [50], [65], which can be obtained from Eqs. (2)–(4) using the correspondence principle. In the resulting classical field approximation, each of the annihilation and creation operators contributes to the correlation functions with the square-root of the intensities of the respective fields, i.e., the field strengths.

This relationship allows us to sort the contributions of the 6 independent Liouville pathways by the powers of their field strengths and their signatures in the electron yield in momentum space (Figure 2E). The second-order peaks at **K** = ±2**k**_SPP_ exclusively consist of a contribution proportional to $E_{\text{SPP}}^{2}E_{\text{Probe}}^{2}$ . The first-order peaks at **K** = ±**k**_SPP_, however, originate from two contributions: one is proportional to $E_{\text{SPP}}E_{\text{Probe}}^{3}$ and the other one is proportional to $E_{\text{SPP}}^{3}E_{\text{Probe}}$ . The situation is even more complicated for the central peak at **K** = 0: it consists of three contributions, proportional to $E_{\text{SPP}}^{4}$ , $E_{\text{Probe}}^{4}$ , and $E_{\text{SPP}}^{2}E_{\text{Probe}}^{2}$ . Note that the contributions proportional to $E_{\text{SPP}}^{4}$ and to $E_{\text{Probe}}^{4}$ are not shown in Figure 2C–F as these only correspond to spatially broad plasmoemission [23] and photoemission backgrounds, respectively.

**Figure 3:**
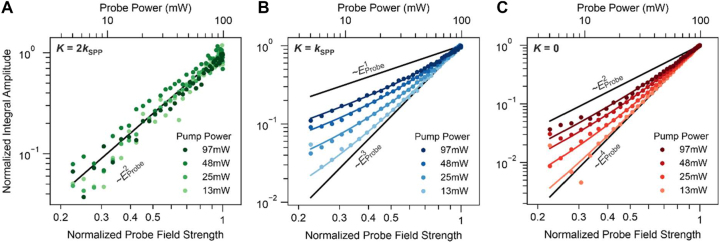
Separation of wavevector peak contributions. (A) Integral amplitude of the second-order wavevector peak at **K** = ±2**k**_SPP_, (B) the first-order wavevector peak at **K** = ±**k**_SPP_, and (C) the central wavevector peak at **K** = 0 as a function of the normalized probe field strength for four different pump powers, plotted on a double logarithmic scale. The measurements are fitted well by the polynomials expected from Eq. (4). As a guide to the eye, the limiting monomial contributions to the expected polynomials are depicted by the straight black lines.

The different scaling behavior of the Liouville pathways with the field strengths provides a means to disentangle them experimentally. We systematically change the pump and probe powers to independently control the absorption probability from the SPP field and the probe laser field, respectively. This procedure provides information on which of the two fields and pathways dominate the electron emission. Figure 3 shows data points of the measured integral amplitude of each wavevector peak as a function of the normalized probe field strength, i.e., the normalized square-root of the probe power. We repeated the measurement for 4 representative pump powers to vary the field strength of the SPP. Each of the curves was normalized to its maximal integral amplitude. The results are plotted on a double-logarithmic scale in Figure 3 such that power laws would appear as straight lines with respective slopes. The solid colored lines in Figure 3 are fits to the data points using the polynomials expected from Eq. (4) combined with the correspondence principle as discussed above. In Figure 3A, the integral amplitude of the second-order peaks at **K** = ±2**k**_SPP_ depends for all pump powers on the normalized probe field strength as $E_{\text{SPP}}^{2}E_{\text{Probe}}^{2}$ , which again suggests that higher emission orders and thus higher-order interactions of the electron system with the SPP and probe fields are negligible. Figure 3B shows the integral amplitude of the first-order peaks at **K** = ±**k**_SPP_. We find that for low pump powers, i.e., weak SPP excitation, the amplitude is dominated by the contribution proportional to $E_{\text{SPP}}E_{\text{Probe}}^{3}$ with a slope of three. This characteristic motivates the recently reported vector microscopy [46]. For high pump powers, i.e., strong SPP excitation, the amplitude in Figure 3B becomes dominated by the contribution proportional to $E_{\text{SPP}}^{3}E_{\text{Probe}}$ with a slope of one. Figure 3C shows the results for the central wavevector peak at **K** = 0. As this central peak also contains all long-range background modulations of the TR-PEEM images, such as plasmo- and photoemission backgrounds, we subtracted a probe-power–independent constant from each of the curves in Figure 3C (see Supplementary Note 2 for details). Note that this subtracted constant includes the contribution proportional to $E_{\text{SPP}}^{4}$ . For low pump powers, i.e., weak SPP excitation, the amplitude of the central peak is dominated by the contribution proportional to $E_{\text{Probe}}^{4}$ . As we increase the pump power, the amplitude of the central peak becomes dominated by the contribution proportional to $E_{\text{SPP}}^{2}E_{\text{Probe}}^{2}$ .

In a classical particle picture, one would expect the probability for an electron to absorb a photon or an SPP to depend on the intensities of the respective fields, which are proportional to the squared magnitudes of the field strengths. This expectation implies it must be possible to attribute a number of absorbed quanta from each of the involved fields to every liberated electron. However, in this classical particle picture, it is difficult to interpret the experimental existence of contributions to the electron yield like $E_{\text{SPP}}E_{\text{Probe}}^{3}$ that scale as odd powers of the field strengths. Such difficulties are not encountered in the quantum analysis leading to Eq. (4).

**Figure 4:**
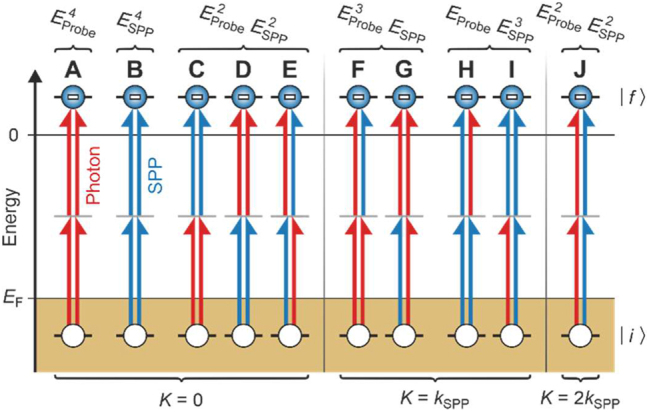
Microscopic picture of the quantum pathways during electron emission. An electron is liberated from an initial state |*i*⟩ below the Fermi energy *E*_F_ to a final state |*f*⟩ in the vacuum by a fourth-order interaction with probe photons (red arrows) and SPPs (blue arrows). In each of the diagrams, the consecutive interactions given by the two arrows on the left-hand side interfere with the consecutive interactions given by the arrows on the right-hand side of that same diagram. The diagrams are grouped by their dependence on the strength of the SPP and the probe field and by their wavevector contribution.

After having demonstrated the existence of all Liouville pathways in Eq. (4) in the experimental data, we now identify which of these pathways must be interpreted as quantum path interferences that arise from the interference of the SPP and photon absorption processes in Figure 1C. All Liouville pathways for the electron emission process in Eq. (4) (except for complex conjugate pathways) are summarized in analogy to double-sided Feynman diagrams [40] in Figure 4. Each of the pathways in Figure 4 consist of four arrows, where the colors red and blue represent an interaction with the probe field and SPP field, respectively. The arrows on the left side of each pathway correspond to creation operators, and the ones on the right side correspond to annihilation operators. Each of the depicted Liouville pathways is associated with a momentum equal to the momentum difference between the left- and right-hand side, which gives rise to the respective peaks in the wavevector spectrum in Figure 2D.

The central wavevector peak at **K** = 0 arises from electron emission by the consecutive absorption of two probe photons (pathway (A)), the consecutive absorption of two SPP quanta (pathway (B)), as well as cooperative pathways (C)–(E). While pathway (C) and (D) correspond to the consecutive absorption of each an SPP quantum and a probe photon, pathway (E) corresponds to the mixing of the consecutive interaction with an SPP quantum and a probe photon (left side of the diagram) and the respective inversely ordered interactions (right side of the diagram), which has been coined nonoscillatory double-mixing [52]. It is worth noting, however, that only within the approximations we applied to the electron system (Supplementary Note 1), the pathways (C), (D), and (E) are physically equivalent, as they can only then be transformed into each other via trivial equal-time commutations of the creation or annihilation operators. Thus, all Liouville pathways that contribute to the central wavevector peak only depend on the SPP and probe photon populations.

The remaining Liouville pathways (F)–(J) cannot be explained by the simple consecutive absorption of SPPs and probe photons. Instead, each of these diagrams must fundamentally be interpreted as a quantum path interference that consist of the interference of electron emission by the consecutive interactions on the left side of a diagram with electron emission by the consecutive interactions on the right side of that same diagram. In the probe-dominated pathways (F) and (G), the consecutive interaction with two probe photons interferes with the consecutive interaction with an SPP and a probe photon. This situation is reversed for the SPP-dominated pathways (H) and (I), where instead the consecutive interaction with two SPPs interferes with the consecutive interaction of an SPP and a probe photon. Moreover, the second-order wavevector peak at **K** = ±2**k**_SPP_, i.e., pathway (J), consists exclusively of the interference of the consecutive interaction with two SPPs and the consecutive interaction with two probe photons. The single-mixing pathways of the first-order wavevector peak and the double-mixing pathway of the second-order wavevector peak probe the mutual first and second-order coherences of the SPPs and of the probe photons, respectively.

Some of the discussed Liouville pathways correspond to observable quantum path interferences in the electron emission – a consequence of the nonlinear mixing of different fields in each of the diagrams contributing to the overall electron emission. By utilizing momentum resolution, we could experimentally distinguish which quantum path interferences (Figure 4F–J) contribute to the electron emission. It is important to note, however, that we did not resolve the individual interactions (as in Figure 1C) that constitute the quantum path interferences. Resolving the individual interactions would be the goal of a which-way experiment, and doing so would indeed destroy the observed quantum path interferences.

## 4 Discussion

Quantum path interferences are a manifestation of the inherent quantum nature of fundamental interactions. Our approach to electron emission in the simultaneous presence of SPPs and light confirms that Liouville pathways can be disentangled by their power-dependent contributions in a momentum space that consists of discrete spots. The here presented work is complementary to a recent time-resolved PEEM experiment where contributions to the electron yield by quantum coherent superpositions of few-plasmon Fock states were disentangled and identified with corresponding Liouville pathways [66]. This work and our work were both carried out on Au(111) substrates that do not host long-lived intermediate electronic states such that in our case electronic excitations by the pump pulse could be neglected. At higher excitation strengths and on different sample substrates, contributions by higher-order interactions with a possibly excited electron system could as well be probed and disentangled in time- and power-dependent measurements [67].

Addressing more complex nontrivial quantum correlations between light and SPPs, like in entangled SPP–photon pairs [68], constitutes a natural progression of our work. We believe that interferences between transitions involving additional quantum numbers for the SPPs, such as spin and orbital angular momentum [52], [53], [69], can be studied most effectively in momentum space as well. Ultimately, adding energy resolution and electron momentum resolution to our technique could provide a route to study nontrivial quantum correlations between interacting quantum electrons, quantum light, and quantum SPPs in the future.

## Supplementary Material

Supplementary Material Details
